# Adverse neonatal outcome and veno-arterial differences in umbilical cord blood pH (ΔpH) at birth: a population-based study of 108,629 newborns

**DOI:** 10.1186/s12884-023-05487-8

**Published:** 2023-03-11

**Authors:** Tiia-Marie Sundberg, Nana Wiberg, Karin Källén, Mehreen Zaigham

**Affiliations:** 1 Obstetrics & Gynecology, Lund University and Skåne University Hospital, 205 01 Malmö, Sweden; 2 Department of Clinical Sciences Lund, Lund University, Lund, Sweden; 3 Department of Clinical Sciences Malmö, Lund University, Malmö, Sweden; 4 Department of Obstetrics & Gynecology, Sjaelland University hospital, 4000 Roskilde, Denmark; 5 Institution of Clinical Sciences Lund, Center for Reproductive Epidemiology, Tornblad Institute, Lund University, Lund, Sweden

**Keywords:** Birth, Birth asphyxia, Perinatal asphyxia, Umbilical cord blood gases, Umbilical cord pH, Umbilical artery pH, Adverse neonatal outcome, Neonatal morbidity, Apgar score, Continuous positive airway pressure, Neonatal intensive care unit

## Abstract

**Background:**

Umbilical cord blood gases are routinely used by midwives and obstetricians for quality assurance of birth management and in clinical research. They can form the basis for solving medicolegal issues in the identification of severe intrapartum hypoxia at birth. However, the scientific value of veno-arterial differences in cord blood pH, also known as ΔpH, is largely unknown. By tradition, the Apgar score is frequently used to predict perinatal morbidity and mortality, however significant inter-observer and regional variations decrease its reliability and there is a need to identify more accurate markers of perinatal asphyxia. The aim of our study was to investigate the association of small and large veno-arterial differences in umbilical cord pH, ΔpH, with adverse neonatal outcome.

**Methods:**

This retrospective, population-based study collected obstetric and neonatal data from women giving birth in nine maternity units from Southern Sweden from 1995 to 2015. Data was extracted from the Perinatal South Revision Register, a quality regional health database. Newborns at ≥37 gestational weeks with a complete and validated set of umbilical cord blood samples from both cord artery and vein were included. Outcome measures included: ΔpH percentiles, ‘Small ΔpH’ (10th percentile), ‘Large ΔpH’ (90th percentile), Apgar score (0–6), need for continuous positive airway pressure (CPAP) and admission to neonatal intensive care unit (NICU). Relative risks (RR) were calculated with modified Poisson regression model.

**Results:**

The study population comprised of 108,629 newborns with complete and validated data. Mean and median ΔpH was 0.08 ± 0.05. Analyses of RR showed that ‘Large ΔpH’ was associated with a decreased RR of adverse perinatal outcome with increasing UApH (at UApH ≥7.20: RR for low Apgar 0.29, *P* = 0.01; CPAP 0.55, *P* = 0.02; NICU admission 0.81, *P* = 0.01). ‘Small ΔpH’ was associated with an increased RR for low Apgar score and NICU admission only at higher UApH values (at UApH 7.15–7.199: RR for low Apgar 1.96, *P* = 0.01; at UApH ≥7.20: RR for low Apgar 1.65, *P* = 0.00, RR for NICU admission 1.13, *P* = 0.01).

**Conclusion:**

Large differences between cord venous and arterial pH (ΔpH) at birth were associated with a lower risk for perinatal morbidity including low 5-minute Apgar Score, the need for continuous positive airway pressure and NICU admission when UApH was above 7.15. Clinically, ΔpH may be a useful tool in the assessment of the newborn’s metabolic condition at birth. Our findings may stem from the ability of the placenta to adequately replenish acid-base balance in fetal blood. ‘Large ΔpH’ may therefore be a marker of effective gas exchange in the placenta during birth.

**Supplementary Information:**

The online version contains supplementary material available at 10.1186/s12884-023-05487-8.

## Introduction

Umbilical cord blood gases are the most objective measure of the newborn’s metabolic condition at birth [1–3]. Sustained intrapartum hypoxia may lead to metabolic acidosis in the fetus and increase the risk of hypoxic brain injury [1, 4]. Previous studies have shown that analysis of cord blood gases, especially cord arterial pH (UApH), can identify newborns at risk of adverse neurological outcomes when most clinical and radiological signs are still absent [3–5]. Cord blood gases are also routinely used in quality assurance of obstetric care by midwives and clinicians, in research and form the basis for solving medicolegal issues in identification of severe intrapartum hypoxia [6].

For the identification of an accurate UApH value, it is important to obtain a correct cord venous pH (UVpH) [7], and although cord arterial blood gases best represent the newborn’s metabolic condition, it is often the umbilical vein that is more easily sampled due to its larger size. Studies have demonstrated the usefulness of cord venous pH as a proxy for cord arterial pH [1, 8]. However, the scientific value of veno-arterial differences in cord pH, also known as ΔpH, are largely undetermined.

Delta pH is widely recognized in the clinical setting, but only a handful of studies have attempted to demonstrate its association with fetal outcomes. A small study evaluating the relationship of ΔpH to cardiotocography patterns found no relation between large ΔpH and acute onset acidemia [9]. Belai and colleagues [10] were able to show a weak correlation of ΔpH to Apgar score, seizures, and hypoxic ischemic encephalopathy. Clinicians, on the other hand, have often relied on the use of the Apgar score to predict adverse neonatal outcome in hypoxic newborns [11–15], but there is need to identify more accurate markers for perinatal asphyxia due to large inter-observer and regional variations with the Apgar score [16]. Determination of ΔpH can therefore be a potential tool for the rapid identification of newborns suffering from perinatal asphyxia.

Using a large, population-based cohort, we aimed to explore the association of veno-arterial differences in cord pH (i.e. the difference between UVpH and UApH), namely ΔpH, with adverse neonatal outcome.

## Methods

### Study population and setting

Data was retrieved from the prospectively collected Perinatal Revision South Register, a quality database collecting obstetric and neonatal data from nine hospitals in Southern Sweden between 1995 and 2015. The original dataset comprised of 315,174 singleton live births with information regarding maternal characteristics, birth, perinatal outcomes, and neonatal course. Since some records from the Perinatal Revision South Register database did not contain complete sets of UApH and UVpH values, the material was supplemented with blood gas data extracted from the maternity records at Skåne University Hospital in Malmö (21,119 records supplemented), which is the largest maternity unit in Southern Sweden.

### Study definitions and validation

Only newborns with a complete set of umbilical cord blood samples from both the artery and vein were included in the study. ΔpH was calculated using the equation:

$$\Delta \textrm{pH}=\textrm{UVpH}-\textrm{UApH}$$

**Fig. 1:**
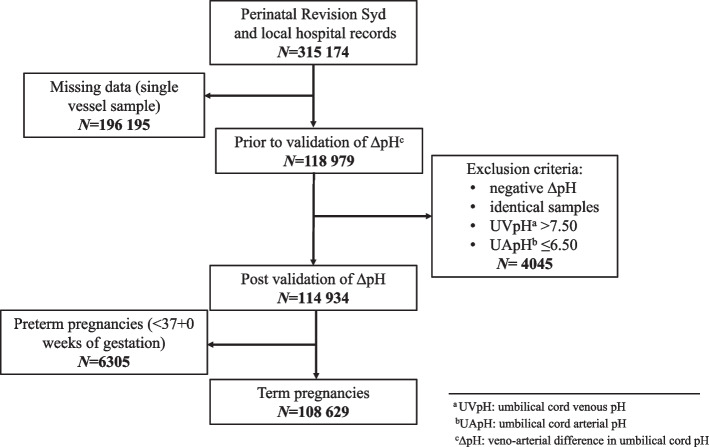
Flow chart showing selection of newborns with paired umbilical artery pH (UApH) and venous pH (UVpH) for calculation of delta pH (ΔpH)

As described by Mokarami et al. [17], both small and negative ΔpH occur physiologically. We therefore validated ΔpH by only removing identical and negative ΔpH values from the dataset to minimize the introduction of selection bias [18]. Similarly, since umbilical cord arterial and venous pH change with advancing gestational age [19, 20], only ‘term’ infants born after 37 completed weeks of gestation were included in the final analyses (*N* = 108,629) (Fig. 1).

Stratification of data were performed as follows: data regarding parity were classified as ‘primipara’, ‘multipara, no previous Cesarean section (CS)’ and ‘multipara, with previous CS’. Similarly, births that were reported to start as CS, but without specified mode of delivery, were regarded as elective CS. Gestational duration was classified term (37 + 0 to 40 + 6 weeks) and (41 + 0 to 41 + 6 weeks) and post-term (≥42 weeks). Birthweight was classified into four groups (< 2500 g, 3000-3999 g, 4000-4499 g, > 4500 g).

In order to study the percentile distribution of ΔpH in relation to changes in cord arterial pH, we divided UApH into six classes (Table 2). Percentiles were for term pregnancies but not parity or fetal sex specific. To calculate the relative risk (RR) for adverse neonatal outcome in association with UApH classes, modified Poisson regression model was chosen. This model was appropriate for large cohorts where independent and rare events occurred at a fixed rate and risk for model misspecification existed [21, 22]. Neonatal outcome parameters included Apgar score (0–6), neonatal intensive care unit (NICU) admission and use of continuous positive airway pressure (CPAP). Finally, to calculate and compare the relative risks of adverse neonatal outcomes, we defined ‘Small ΔpH’ as all ΔpH values less than or equivalent to 0.02 (10th percentile, or ≤ 0.02), and defined ‘Large ΔpH’ as all ΔpH values greater or equivalent to 0.15 (90th percentile, or ≥ 0.15). These cut-off values were selected to elucidate the adverse neonatal outcomes associated with extremes of ΔpH i.e., when ΔpH was assumed to be no longer ‘normal’.

### Data management and analysis

Statistical analyses were performed with SPSS data software (26) (IBM Corp, Armonk, NY). Background characteristics were calculated and distributions between parameters were compared with Pearson Chi-2-test. The distribution of ΔpH across the population was illustrated by histogram to investigate normality and percentile distribution of ΔpH in different UApH groups. Modified Poisson regression model with 95% confidence intervals (CI) was used for RR of adverse neonatal outcomes.

## Results

### Characteristics of the study population

The selection of newborns for inclusion in the study is illustrated in Fig. 1. From the initial 315,174 newborns, after strict validation and exclusion of all preterm cases, 206,545 were excluded. The final study population comprised of 108,629 newborns with complete and validated data. Background characteristics are displayed in Table 1. The study population was described and cut-offs for ‘Small ΔpH’ (10th percentile, ≤0.02) and ‘Large ΔpH’ (90th percentile, ≥0.15) were also described. Vaginal non-instrumental birth (78.4%) was associated with higher rate of ‘Large ΔpH’ (87.7%) as compared to ‘Small ΔpH’ (75.9%). Neonates with birthweight < 2500 g (0.9%) accounted for 1.5% of all neonates who had a ‘Small ΔpH’. In contrast, birthweight 4000-4499 g (16.2%) and 4500 g + (4.1%) had higher percentage of neonates with ‘Large ΔpH’ (21.6 and 5.8%). Using the Chi-2-test, differences between ‘Small’ and ‘Large ΔpH’ were compared and found to be statistically significant for maternal age, parity, maternal body mass index (BMI), maternal smoking, birth start, birth mode, gestational age, birthweight, and Apgar score at 5 minutes. Infant sex was therefore the only background characteristic that was evenly distributed between the two cut-off groups.

**Table 1:** Background characteristics of the study population including Chi-2-test comparing ‘Small ΔpH’ (10th percentile ≤0.02) and ‘Large ΔpH’ (90th percentile ≥0.15)

	*N*	%	Small ΔpH^a^ ≤ 0.02		Large ΔpH ≥0.15		Chi-2-test
			*N*	%	*N*	%	*P-*value
Total *N*	108,629	100	12,015	100	12,261	100	
Maternal age in years							0.028
< 20	2053	1.9	229	1.9	184	1.5	
20–34	85,681	78.9	9543	79.4	9671	78.9	
35–39	17,552	16.2	1904	15.8	2050	16.7	
40+	3343	3.1	339	2.8	356	2.9	
Parity							< 0.000
Primipara	50,116	46.1	5867	49.2	5159	42.6	
Multipara	57,372	52,8	6054	50.8	6960	57.4	
Multipara with previous CS^b^	5464	5.0	–	–	–	–	
Missing	1141	1.1	–	–	–	–	
Maternal BMI^c^							0.005
< 18.5	149	0.1	25	3.4	11	1.6	
18.5–24.9	3433	3.2	384	51.7	306	45.7	
25–29.9	2221	2.0	247	33.2	243	36.3	
30+	885	0.8	87	11.7	109	16.3	
Missing data	101,941	93.8	–	–	–	–	
Maternal smoking							< 0.000
No	70,714	65.1	8099	91.5	7898	93.6	
Yes 1–9 cigarettes/day	4635	4.3	551	6.2	400	5.5	
Yes, ≥10 cigarettes/day	1718	1.6	119	2.2	139	2.0	
Missing data	31,562	29.1	–	–	–	–	
Birth start							< 0.000
Spontaneous	91,017	83.8	10,020	83.4	10,885	88.8	
Induction	10,379	9.6	1183	9.9	1242	10.1	
CS	7214	6.6	807	6.7	132	1.1	
Missing data	19	0.0	–	–	–	–	
Birth mode							< 0.000
Vaginal, non-instrumental	85,196	78.4	9052	75.9	10,636	87.7	
Elective CS	6623	6.1	713	6.0	118	1.0	
Emergency CS	7785	7.2	1167	9.8	112	0.9	
Immediate emergency CS	791	0.7	155	1.3	43	0.4	
Instrumental (VE^d^/forceps)	7407	6.8	837	7.0	1215	10.0	
Missing	827	0.8	–	–	–	–	
Gestational duration in weeks (days)							< 0.000
37 + 0–40 + 6 (259–286)	80,716	74.3	9117	75.9	8780	71.6	
41 + 0–41 + 6 (287–293)	20,072	18.5	2038	17.0	2531	20.6	
> 42 + 0 (294+)	7841	7.2	860	7.2	950	7.7	
Sex							0.132
Male	56,300	51.8	6148	51.2	6432	52.5	
Female	52,244	48.1	5855	48.7	5817	47.4	
Missing	85	0.1	12	0.1	12	0.1	
Birthweight (g)							< 0.000
< 2500	931	0.9	177	1.5	26	0.2	
2500–3999	85,612	78.8	9731	81.0	8880	72.4	
4000–4499	17,569	16.2	1703	14.2	2647	21.6	
4500+	4463	4.1	397	3.3	706	5.8	
Missing data	54	0.0	–	–	–	–	
Apgar score at 5 minutes							0.001
0–3	95	0.1	9	0.1	21	0.2	
4–6	863	0.8	109	0.9	161	1.3	
7–10	107,652	99.1	11,895	99.0	12,078	98.5	
Missing data	19	0.0	–	–	–	–	

^a^Δ*pH* veno-arterial differences in umbilical cord pH, ^b^ *CS* Cesarean section, ^c^ *BMI* body mass index (kg/m^2^), ^d^ *VE* vacuum extraction

### ΔpH distribution

**Fig. 2:**
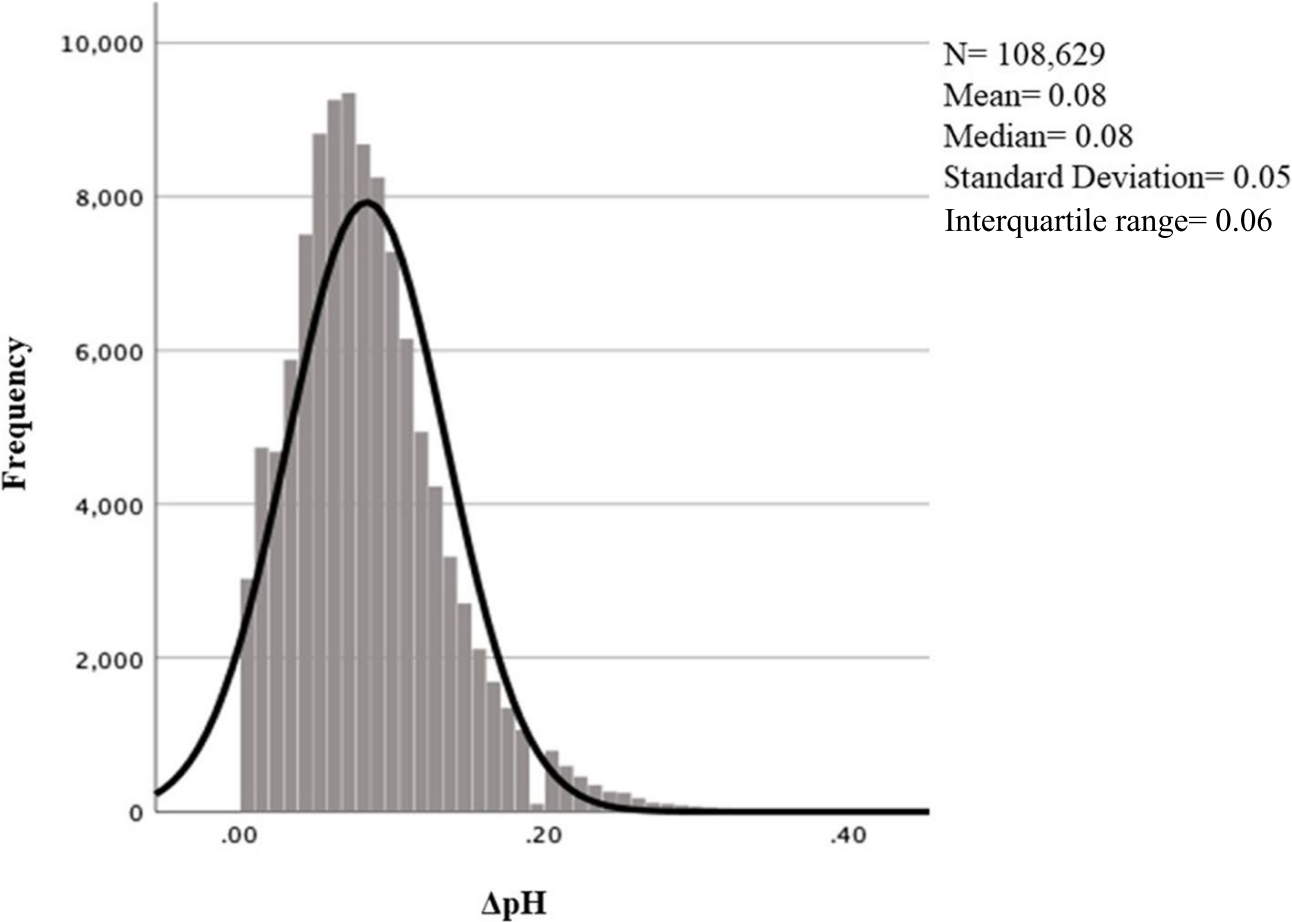
Histogram with distribution of veno-arterial differences in umbilical cord pH (ΔpH) in term pregnancies after validation

The distribution of ΔpH across the study population is illustrated in Fig. 2. Both the mean and median ΔpH were 0.08, standard deviation 0.05 and interquartile range 0.06. In Table 2, percentile values including median and mean values for ΔpH are reported for the whole study population (*N* = 108,629) and for UApH classes. The mean ΔpH decreased with rising UApH, from 0.13 in pH < 7.00 to 0.07 in pH ≥7.20 and varied within each percentile.

**Table 2:** Percentile distribution of ΔpH in relation to umbilical cord arterial pH (UApH) in term pregnancies (N = 108,629)

	*Number of cases*	ΔpH percentiles							
		5%	10%	25%	Median	75%	90%	95%	Mean
Total cases	**108,629**	**0.01**	**0.02**	**0.05**	**0.08**	**0.11**	**0.15**	**0.18**	**0.08**
UApH^a^									
< 7.00	799	0.02	0.03	0.06	0.10	0.18	0.29	0.35	0.13
7.00–7.049	1405	0.02	0.04	0.07	0.12	0.19	0.26	0.31	0.14
7.05–7.09	3466	0.02	0.04	0.07	0.12	0.17	0.23	0.26	0.13
7.10–7.149	8345	0.02	0.04	0.07	0.11	0.16	0.20	0.23	0.12
7.15–7.19	15,920	0.02	0.04	0.07	0.10	0.14	0.18	0.20	0.11
≥ 7.20	78,694	0.01	0.02	0.04	0.07	0.10	0.13	0.15	0.07

^a^ *UApH* umbilical cord arterial pH

### ΔpH and adverse neonatal outcomes

**Fig. 3:**
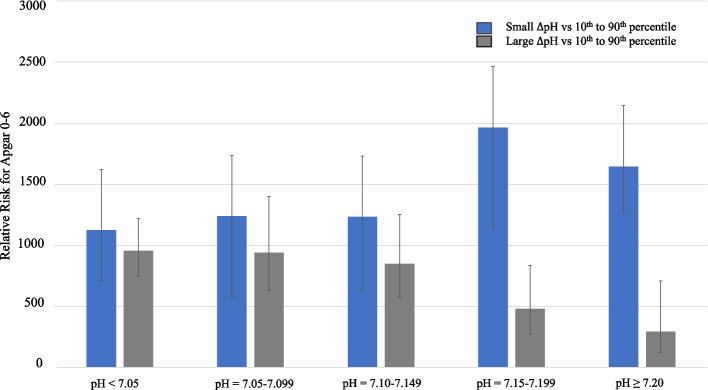
Correlation of ΔpH to relative risk of 5-minute Apgar score 0–6 and changes in umbilical cord arterial pH

In Table 3, the RR for ‘Small’ and ‘Large ΔpH’ were compared to ΔpH between the 10th and 90th percentiles and parameters associated with adverse neonatal outcomes. For ‘Small ΔpH’, at UApH < 7.15–7.199, the RR for Apgar 0–6 at 5 minutes was RR = 1.96 (95%CI = 1.15–3.37) (*P* = 0.01); and at UApH ≥7.20, the RR was 1.65 (95%CI = 1.26–2.1) (*P* < 0.00). In contrast, a lower relative risk was found for ‘Large ΔpH’ for Apgar 0–6 at 5 minutes. At UApH 7.15–7.199, the RR was 0.48 (95%CI = 0.27–0.84) (*P* = 0.01). This apparent protective effect was enhanced for UApH ≥7.20, where the RR for Apgar 0–6 was 0.29 (95%CI = 0.12–0.71) (*P* = 0.01). These changes are illustrated in Fig. 3, where the RR for Apgar 0–6 is seen to decrease with enhancing UApH for ‘Large ΔpH’.

**Table 3:** Relative risk of adverse neonatal outcome with Small ΔpH (≤0.02; 10th percentile) or Large ΔpH (≥0.15; 90th percentile) as compared to ΔpH between 10th–90th percentiles. Modified Poisson regression model with 95% confidence intervals and *P*-values were calculated for term pregnancies. N = 108,629

	Total cases	108,629	%	Apgar 0–6 at 5 minutes			CPAP^**a**^			NICU^**b**^ admission		
				*P*-value	RR ^c^	95% CI^d^	*P*-value	RR	95% CI	*P*-value	RR	95% CI
**UApH < 7.05**	Small ΔpH	135	6.1%	0.62	1.12	0.71–1.77	0.457	0.800	0.44–1.44	0.89	0.98	0.74–1.30
	Large ΔpH	813	36.9%	0.71	0.95	0.75–1.22	0.199	0.833	0.63–1.10	0.15	0.90	0.78–1.04
	Total	2204	100%									
**UApH 7.05–7.099**	Small ΔpH	178	5.1%	0.58	1.24	0.58–2.66	0.404	0.708	0.32–1.59	0.71	0.93	0.64–1.36
	Large ΔpH	1208	34.9%	0.76	0.94	0.63–1.40	0.082	0.730	0.51–1.04	0.00	0.75	0.62–0.91
	Total	3466	100.0%									
**UApH 7.10–7.149**	Small ΔpH	456	5.5%	0.55	1.23	0.62–2.44	0.380	0.728	0.36–1.48	0.36	1.14	0.86–1.50
	Large ΔpH	2577	30.9%	0.41	0.85	0.57–1.25	0.924	1.015	0.75–1.37	0.24	0.91	0.79–1.06
	Total	8345	100.0%									
**UApH 7.15–7.199**	Small ΔpH	901	5.7%	0.01	1.96	1.15–3.37	0.411	0.755	0.39–1.47	0.99	1.00	0.78–1.28
	Large ΔpH	3452	21.7%	0.01	0.48	0.27–0.84	0.048	0.679	0.46–1.00	0.01	0.82	0.70–0.95
	Total	15,920	100.0%									
**UApH ≥ 7.20**	Small ΔpH	10,345	13.1%	0.00	1.65	1.26–2.1	0.918	1.013	0.78–1.31	0.01	1.13	1.04–1.24
	Large ΔpH	4211	5.4%	0.01	0.29	0.12–0.71	0.022	0.549	0.30–0.92	0.01	0.81	0.69–0.95
	Total	78,694	100.0%									

^a^ *CPAP* continuous positive airway pressure, ^b^ *NICU* neonatal intensive care unit, ^c^ *RR* relative risk, ^d^ *CI* confidence interval

Of interest, ‘Large ΔpH’ showed the same protective trend at UApH 7.15–7.199 from need of CPAP (*P* = 0.048) and NICU admission (*P* = 0.01); and at UApH ≥7.20 for CPAP (*P* = 0.02) and NICU admission (*P* = 0.01).

## Discussion

We investigated the association of veno-arterial differences in cord pH (ΔpH) with adverse neonatal outcome and found that large differences between umbilical cord venous and arterial pH (‘Large ΔpH’) were strongly associated with a lower relative risk for Apgar < 7 at 5 minutes, need for CPAP and NICU admission. These changes were strongest for infants born without acidemia.

As oxygen, carbon dioxide and hydrogen ions readily diffuse across the placenta to the maternal side for elimination, the fetus is dependent on adequate placental function in order to maintain its acid-base balance [23]. This dependency is especially important during birth, when the intermittent compressions of the umbilical cord, caused by forceful uterine contractions, lead to partial suspension of cord blood flow to and from the fetus leading to considerable metabolic stress in the fetus [24].

Clinically, most cases of fetal acidosis are acute in onset and are accompanied by large veno-arterial differences in pH (i.e. ‘Large ΔpH’). This is because the extracellular placental compartment has not had enough time to become saturated with acids from the fetus, or cord occlusion or fetal bradycardia have prevented fetal acids from being transferred to the placenta [7, 25, 26]. However, where a degree of chronic hypoxia has occurred, both the venous and arterial gases will show metabolic acidosis with a small veno-arterial difference (i.e. ‘Small ΔpH’) [7, 25, 26]. This information explains our finding that ‘Large ΔpH’ were associated with a decreased risk of adverse neonatal outcome because it likely reflects an acute intrapartum insult with a venous pH in the normal range.

Earlier studies have investigated the role of different cord blood gas parameters in predicting perinatal outcomes. Knutzen et al. [27] found that once pH was considered, base deficit was not of additional help in predicting adverse neonatal outcomes. As base deficit is a key component in respiratory acidosis, Low et al. [28] showed that acute acidosis was not linked to adverse outcome in the same way as metabolic acidosis. On the other hand, Wiberg and colleagues [29] found that umbilical cord lactate values were as good as cord pH or base deficit in predicting depressed neonatal condition at birth. Due to methodological confounding involved in calculation of base deficit, lactate may replace base deficit as an acid–base outcome parameter at birth. In essence, these studies highlight the difference between respiratory acidosis, which is considered short standing, as compared to the more longstanding metabolic acidosis that depletes the fetus’s buffer store.

It is well known that umbilical cord pH analysis gives a picture of the metabolic state of the newborn at birth, but previous studies have presented conflicting data. Martin et al. [30] in a small study comparing nuchal cord labors with acidosis (*N* = 33) to normal labors, found large differences between UVpH and UApH but the Apgar score was not affected. Different outcomes have been observed for acute versus the chronic acidosis, as seen by Bobrow et al. [24], where chronic acidosis was associated with adverse outcome for neonates. Importantly, since pH is based on a logarithmic scale, a unit decrease in pH is much more substantial than a unit decrease in the corresponding hydrogen ion concentration, an effect that becomes ever stronger with decreasing pH [31].

Our data showed that a ‘Large ΔpH’ was associated with decreased risk for adverse neonatal outcome, yet only 1 % of the cases with the lowest Apgar score had a ‘Small’ or ‘Large ΔpH’.

Whilst reviewing the literature, only a handful studies were found to have investigated the prognostic value and clinical significance of veno-arterial differences in cord pH [1, 4, 8–10, 29] with only one study correlating these differences to adverse neonatal outcome. A study evaluating ΔpH in relation to cardiotocography patterns, found no relation between large ΔpH and acute onset acidemia [9]. The sample size, however, was small (*N* = 83). Similarly, Belai et al. [10], showed a weak correlation of ΔpH to Apgar Score, seizures and hypoxic ischemic encephalopathy. By using a large population-based cohort with validated cord blood gases, this study has comprehensively correlated differences in ΔpH to adverse neonatal outcome.

The major strengths of the current study stem from the large sample size and the meticulous validation of paired umbilical cord blood pH values. As small ΔpH may occur physiologically [17], cut-offs were set at identical and negative ΔpH to minimize the introduction of selection bias. There are several pitfalls in cord blood sampling and analyses [17], but the procedure has been routinely used in Sweden for many years and a high-quality dataset was therefore obtained [32]. On the other hand, discarding all single pH samples could have introduced a systematic selection bias in the study. Possible causes for single vessel samples, at 62% of all cases can be explained in several clinical situations. Dual vessel samples are generally drawn from the cord at birth but may not be analyzed when the newborn is healthy. In contrast, with an emergency birth or a suspected asphyxiated newborn, umbilical cord blood gases may not be prioritized due to the urgency in the care of the infant and may lead to a single sample taken to save valuable time. Regardless, the sample size of the current study was large enough to account for this step in the data cleaning process which was essential to obtain values from both cord artery and vein pH. Data was obtained from an established regional database which was used by nine different maternity centers all following the same general routines and procedures. The study population was therefore representative of the whole population [32].

## Conclusion

Large ΔpH was associated with a lower risk for neonatal morbidity including low 5-minute Apgar score, the need for CPAP and NICU admission when UApH was above 7.15. Reference values for ∆pH, including stratification of percentile values according to UApH, were reported. Our findings may have important clinical implications in the assessment of the metabolic state of the newborn at birth. They may stem from the positive effects of maintained placenta function during birth and the ability of the placenta to adequately replenish acid-base balance in fetal blood leading to a large veno-arterial differences in cord blood pH. A ‘Large ΔpH’ may therefore be a marker of effective gas exchange in the placenta during birth. Further studies are warranted to investigate changes in ΔpH in correlation to carbon dioxide and oxygen levels in umbilical cord blood gases to verify this hypothesis.

## Supplementary Information

**Additional file 1.** Correlation of ΔpH decentiles to relative risk of 5-minute Apgar score 0–6.

## Data Availability

The datasets generated and analyzed during the current study are not publicly available due to agreements signed with the Regional Ethics Committee in Lund under the Ethical Approval of the study but are available from the corresponding author on reasonable request.

## Abbreviations

ΔpH: delta pH
UVpH: umbilical cord venous pH
UApH: umbilical cord arterial pH
CPAP: continuous positive airway pressure
NICU: neonatal intensive care unit
RR: Relative risk
