# Supplementary material for: Adverse neonatal outcome and veno-arterial differences in umbilical cord blood pH (ΔpH) at birth: a population-based study of 108,629 newborns
Source: BMC Pregnancy Childbirth. 2023 Mar 11;23:162. doi: 10.1186/s12884-023-05487-8 (PMC10007827; doi:10.1186/s12884-023-05487-8)
Supplement: Supplementary file 1 — Additional file 1. Correlation of ΔpH decentiles to relative risk of 5-minute Apgar score 0–6. [file 12884_2023_5487_MOESM1_ESM.pptx]

## Slide 1
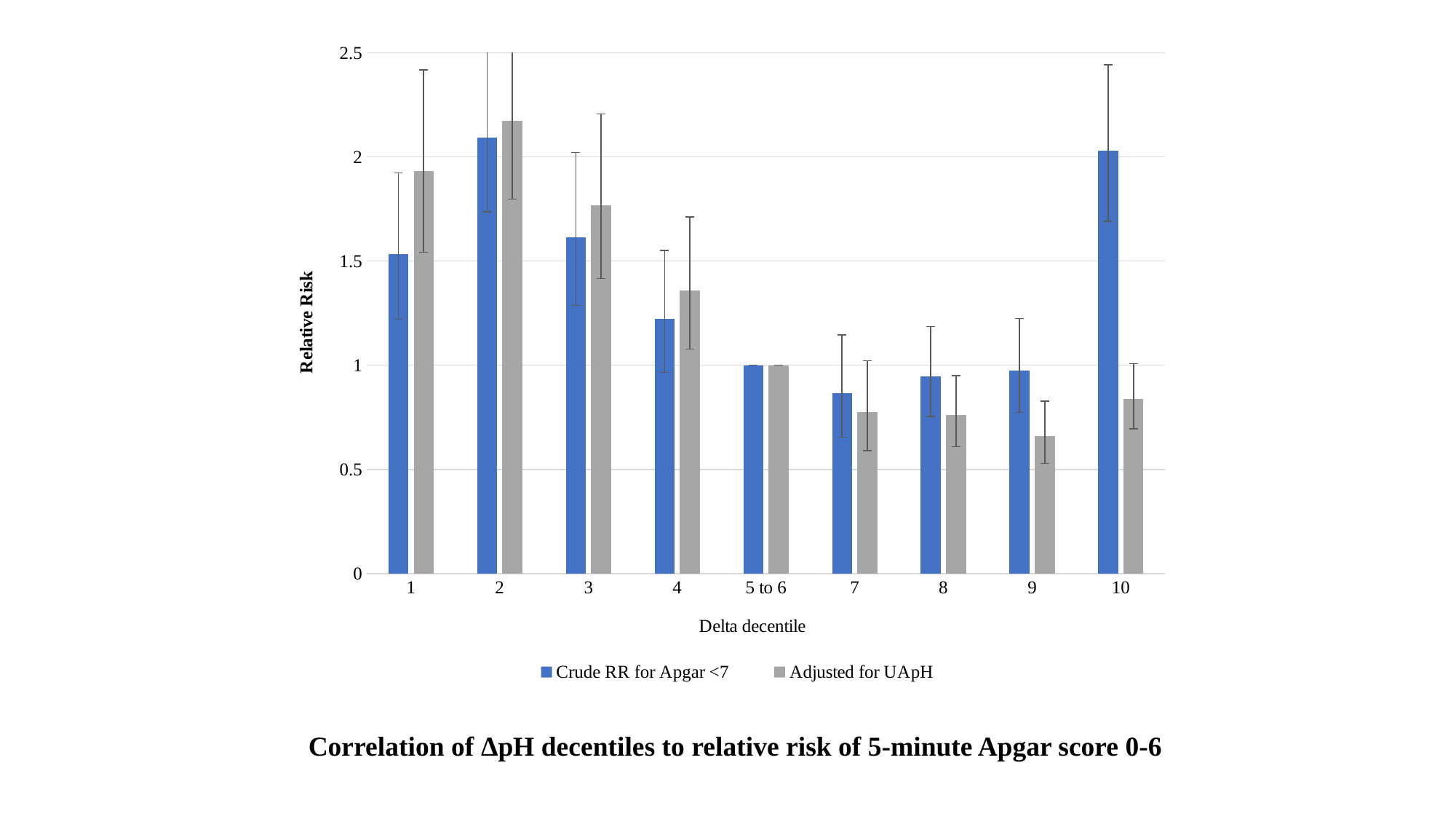

### Chart
| Category | Crude RR for Apgar <7 | Adjusted for UApH |
|---|---|---|
| 1 | 1.532450364209798 | 1.931000661626765 |
| 2 | 2.091125885240341 | 2.1737141731924936 |
| 3 | 1.6134233824987452 | 1.7681152823703359 |
| 4 | 1.2243463211002408 | 1.3584627804548774 |
| 5 to 6 | 1.0 | 1.0 |
| 7 | 0.8659224811142713 | 0.7768990282354805 |
| 8 | 0.9460779314656599 | 0.7615091112306905 |
| 9 | 0.9728233871077262 | 0.6616585609718405 |
| 10 | 2.031191105280555 | 0.8371148038451439 |Correlation of ΔpH decentiles to relative risk of 5-minute Apgar score 0-6
